# Editorial: Plant response to high ambient temperature

**DOI:** 10.3389/fpls.2022.971480

**Published:** 2022-07-18

**Authors:** Ziqiang Zhu, Lam Dai Vu, Sureshkumar Balasubramanian

**Affiliations:** ^1^School of Life Sciences, Nanjing Normal University, Nanjing, China; ^2^Department of Plant Biotechnology and Bioinformatics, Ghent University, Ghent, Belgium; ^3^VIB Center for Plant Systems Biology, Ghent, Belgium; ^4^School of Biological Sciences, Monash University, Clayton, VIC, Australia

**Keywords:** high temperature response, phosphoproteomics, chloroplast biogenesis, phospholipids, *Serendipita indica*, *Agrostis stolonifera*, *Brassica napus*, *Triticum aestivum*

With global warming, there is an urgent need to understand how plants adapt to high temperature. It has been estimated that the global crop yield will be dramatically decreased in the future if we cannot generate heat tolerant crop varieties. For highlighting new discoveries in the field of plant response to high temperature, we organize this timely Research Topic in the Frontiers in Plant Science. This Research Topic on *Plant Response to High Ambient Temperature* comprises 8 original research articles. Although mechanistic studies are mostly from research performed in *Arabidopsis thaliana*, this topic has a broad scope and therefore includes work across diverse plant species, including *Begonia grandis, Brassica napus, Agrostis stolonifera* and *Triticum aestivum*.

Thermomorphogenesis refers to morphological alterations induced by temperature change. Typically this refers to changes observed when plants are grown under high ambient temperature conditions. Plants display elongated hypocotyls, petioles and leaf hyponasty for cooling their leaf surfaces. Transcription factor PIF4 is the central regulator for thermomorphogenesis. Hwang et al. reveals that B-box zinc finger protein BBX18 interacts with PRR5 protein, which represses *PIF4* transcription. The interaction between BBX18 and PRR5 reduces the inhibition on *PIF4*. The authors further show that overexpression of *BBX18* stimulates thermomorphogenesis in a PIF4-dependent manner. In addition to the regulation of plant architecture, temperatures also modulate plant organelle functions. Li et al. identifies a temperature sensitive mutant called *thermos-sensitive mutant in leaf color 2* (*tsl2*). The *tsl2* mutants exhibit abnormal chloroplast development at 16°C but show normal chloroplasts at 29°C. Bulk Segregant Analysis studies clone the *TSL2* gene, which encodes FtsH-Inactive Protein 5 (FtsHi5). The authors further substantiate the connection between FtsHi5 and chloroplast proteome under different temperatures.

Although there are plenty of progress in the area of plant temperature sensing and signaling, there are still a lack of systematic analysis, especially at the proteomic level. In this issue, Shao et al. generates a high quality phosphoproteomics resource for the community. They identify 13160 phosphopeptides in 5125 proteins in high temperature treated samples. Among these phosphorylated proteins, 180 proteins are upregulated and 87 proteins are downregulated by high temperature. The authors also check the protein stability in one differentially phosphorylated protein (ATL6) to prove the concepts of their findings. Researchers who are interested in protein phosphorylation are encouraged to look at this dataset. Meanwhile, Sun et al. studies lipidomic changes in *Begonia grandis* under heat stress. They find that under heat stress, three types of triacylglycerols (18:0/16:0/16:0, 16:0/16:0/18:1, and 18:3/18:3/18:3) are induced, but lysophospholipids and sphingolipids are reduced. These two pieces of large-scale studies shed new light in our understanding of plant responses in the proteome and lipidome to high temperatures.

Researches in plant response to high temperatures aim to understand the consequences of heat on crop yield. Macova et al. investigates the impact of high temperatures on seed development in *Brassica napus* (the second most important oilseed crop). They report that high temperatures cause lower fertilization rates, defective embryonic development, altered glucosinolate contents and oil composition and thus, reduced seed dormancy and seed quality. These physiological responses would be a potential focus in future research to improve the quality of rapeseeds or other important crop seeds. Lima et al. compares the agronomic traits in 12 high-yielding European bread wheat varieties in the Mediterranean rainfed conditions. Their results provide a resource for cultivar selections in specific regions. With similar strategies, Li et al. measures physiological traits in 42 accessions of creeping bentgrass (*Agrostis stolonifera*) to assess their heat tolerance in both growth chambers and field during summer. According to their report, the 13M, PROVIDENCE and LOFTS L-93 accessions exhibit superior tolerance to heat than other materials because these lines have better reactive oxygen species scavenging capacity and higher endogenous gamma-aminobutyric acid levels.

Last but not least, plants are not living alone. In natural, endophytic fungi symbiotic promotes plant growth and enhances fitness. Chen et al. studies *A.thaliana* responses to beneficial endophytic fungus *Serendipita indica* under high ambient temperatures. They conclude that high temperatures strengthen the growth promotion effect.

These eight original research articles are definitely not the end of research on plant response to high temperature. Scientists and researchers on this field will continue to explore new findings. But we do wish that we can stop or slow down our research on plant responses to high temperature at some day. Because it means at that time, we already archive our goal in the controlling of global warming!

## Author contributions

All authors listed have made a substantial, direct, and intellectual contribution to the work and approved it for publication.

## Funding

This research in ZZ lab was supported by the National Natural Science Foundation of China (31970256). LD was supported by the post-doctoral fellowship of the Special Research Fund (Bijzonder Onderzoeksfonds, Ghent University). Research on temperature in SB Lab was supported by Australian Research Council Discovery Project Grant DP190101818.

## Conflict of interest

The authors declare that the research was conducted in the absence of any commercial or financial relationships that could be construed as a potential conflict of interest.

## Publisher's note

All claims expressed in this article are solely those of the authors and do not necessarily represent those of their affiliated organizations, or those of the publisher, the editors and the reviewers. Any product that may be evaluated in this article, or claim that may be made by its manufacturer, is not guaranteed or endorsed by the publisher.

